# The Examination of the Relationship Between the Number of Births with the Symptoms of Urinary Incontinence and Low Back Pain Postpartum in Greek Women

**DOI:** 10.3390/medsci13010022

**Published:** 2025-03-01

**Authors:** Eleni Katsouli, Eleni-Alexandra Karathanasi, Eleftheria Ntalagianni, Themistoklis-Marios Terpos, Anna Christakou

**Affiliations:** 1 Department of Physiotherapy, School of Health Sciences, University of Peloponnese, 23100 Sparta, Greece; elenakatsouli03@gmail.com (E.K.); karaelena345@gmail.com (E.-A.K.); ellie.ntala@gmail.com (E.N.); themistoklhsterpos@gmail.com (T.-M.T.); 2 Laboratoty of Biomechanics, Department Physiotherapy, School of Health Sciences, University of Peloponnese, 23100 Sparta, Greece

**Keywords:** urinary incontinence, low back pain, postpartum, pregnancy, number of births

## Abstract

Background: Urinary incontinence and low back pain are often present during pregnancy and after childbirth. The aim of this study was to examine the relationship between the number of children with the occurrence of urinary incontinence and low back pain after pregnancy in the Greek population. Materials and Methods: Seventy-one Greek women (M = 35.0 age, SD = ±4.3) with specific inclusion criteria completed just once the International Consultation on Incontinence Questionnaire and the Oswestry Disability Questionnaire after five years from childbirth. Results: A total of 28.2% of the participating women experienced urinary incontinence, and 38% experienced low back pain after pregnancy. No relationship has been found between urinary incontinence and the number of births (r = 0.062, *p* = 0.609) and low back pain with the number of births (r = −0.076, *p* = 0.529). Statistically significant correlations were found between urinary incontinence and low back pain (r = 0.33, *p* < 0.01) and the urinary incontinence and the maternal age at first delivery (r = −0.264, *p* = 0.026) in women who underwent a vaginal delivery in second birth had fewer urinary incontinence symptoms and increased low back pain. Conclusions: Few correlations emerged in the present study. Future research is necessary to be conducted to examine the relationship between postpartum women’s demographic data, urinary incontinence, and low back pain.

## 1. Introduction

Urinary incontinence (UI) is described as the involuntary loss of urine that affects the quality of sleep and physical, sexual, and mental health [1]. Multiple childbirths (number of deliveries ≥ 2), older maternal age (≥35 years), increased body mass index (BMI) before pregnancy, higher weight of the newborn, prior record of UI and pregnancy are significant risk factors for the appearance of UI [2,3,4]. Incident rates during pregnancy and the first year after childbirth range from 22.6 to 66.8% [5] and 6.8 to 33.9%, respectively, as these ranges are influenced by women’s daily lives [6]. Low back pain (LBP) is described as pain or discomfort located between the last lateral rib and the gluteal crease area, with or without pain in the legs [7]. It occurs when the patient comes into anterior flexion with mobility restriction in the lumbar region and is characterized by dull pain [8]. LBP is a common cause of discomfort during pregnancy and postpartum, with around 50% of women reporting pain in the lumbar area [9]. Incidences and episodes of LBP before and during pregnancy are risk factors for postpartum LBP, with symptoms persisting up to fourteen months after childbirth [10,11].

Vaginal delivery has been associated with immediate occurrence of UI symptoms in contradiction to cesarean surgery [12]. A lower prevalence of UI and fewer symptoms during daily and sports activities have been found in caesarean delivery in women weightlifters. Caesarean delivery has also been declared as a protective agent against postpartum UI [2]. Repeated vaginal deliveries and vaginal deliveries performed with assistive equipment affect the function of the pelvic floor, with a higher possibility of UI [13,14]. UI has been associated with the week of gestation and with an increased BMI during pregnancy, according to the International Consultation on Incontinence Questionnaire—Urinary Incontinence Short Form (ICIQ-UI SF) measure [12,15,16]. Although older maternal age is a risk factor for UI [2,3], there is also a correlation between younger maternal age and UI [17].

During pregnancy and four months postpartum, excessive weight gain [18] and reduced trunk muscle strength during the same period [19] have been shown to be associated with painful lower back and pelvic pain [20]. Lumbar pain has been associated with stress urinary incontinence up to three months postpartum [21]. A higher prevalence of chronic LBP has been linked with women with at least one childbirth in comparison with women who have not given birth (nulliparous). There was no difference in comparing the symptoms of LBP between women with one childbirth and women with multiple childbirths [22]. The study examined an association between spinal posture and spinal deformities, and it reported the importance of further analysis of the association between low back pain and the number of births [23]. A previous study investigated the persistence of pregnancy-related LBP (PLBP) symptoms and pelvic girdle pain (PGP) symptoms up to fourteen months postpartum and encouraged the further investigation of the risk factors for postpartum persistence PLBP and PGP [11]. The existing literature shows the incidence of LBP from three months [21] and up to six years [24]; however, there is no study that examines the incidence of LBP and the relationship between LBP and the number of children five years postpartum. There is a strong effect of vaginal delivery on the symptoms of urinary incontinence, particularly after five years of childbirth [25]; however, there is no examination of a correlation between the number of children and the symptoms of UI five years postpartum.

The purpose of the present study was to examine the possible correlations between the number of pregnancies with both UI and LBP five years postpartum in the Greek population. The significance of this study is very important, as it contributes to reducing the postnatal effects in this population, increases functional recovery, and further researches the remaining presence of UI and LBP symptoms five years postpartum.

## 2. Materials and Methods

### 2.1. Study Design

This was a cross-sectional study. This study has been registered and approved by the Ethics Committee of the School of Health Studies of the University of Peloponnese (11097/17-05-2024). The study was in agreement with the declaration of Helsinki Ethics principles.

### 2.2. Participants

A priori power analysis was conducted using G*Power version 3.1.9.7, which showed that we would need at least 69 participants with an 80% power for detecting a large effect and a significance criterion of α = 0.05. Thus, 71 postpartum women with an average age of 35 ± 4.3 years old were recruited from local medical facilities (Table 1).

Inclusion criteria were as follows: (1) women aged 18–49 [12], (2) 5 years after childbirth [25], (3) at least one birthing [22], (4) understanding of the Greek language [26,27]. The exclusion criteria were as follows: (1) neurogenic bladder dysfunction, lack of independent mobility and cognitive impairment [21,28], (2) medication for UI, (3) active urinary tract infection, (4) previous medical intervention or surgery to treat UI, (5) pelvic organ prolapses, (6) chronic orthopedic or inflammatory disorders [20], (7) incontinence prior to pregnancy (8) and pregnancy [28].

### 2.3. Instruments

The instruments were as follows:

- The Greek version of the International Consultation on Incontinence Questionnaire—Urinary Incontinence Short Form (ICIQ—UI SF) [26,29]. It was used to evaluate the presence of UI in postpartum women: The questionnaire assesses the severity of UI symptoms and their impact on an individual’s quality of life. It examines three factors: quantity (rated from 0 to 6), frequency (rated from 0 to 5), and sense of incontinence (rated from 0 to 10). The final score ranges from 0 to 21 and is the sum of all scores. A final score of 1 to 5 is designated as mild incontinence, 6 to 12 as moderate incontinence, 13 to 18 as severe incontinence, and 19 to 21 as very severe incontinence. The last question is not taken into account in the score. We asked them to evaluate their symptoms based on the last four weeks [30]. This questionnaire has been widely used in the Greek population [28].
- The Greek version of the Oswestry Disability Questionnaire (ODQ) [27,31]. It was used to assess low back pain, which consists of 10 questions concerning patients with acute and chronic low back pain. The questions examine the degree of disability and present a percentage rating of the level of functioning in daily activities. The scale is six-point, with 0 corresponding to “no” LBP and 5 to “maximum” LPB. The total score is 100%, indicating the highest degree of disability. Factors examined were pain intensity, personal care, weight lifting, walking, sitting, standing, sleeping, sex life, social life, and travel. This questionnaire has been used in the Greek female population [32,33].

### 2.4. Procedure

Participants provided written, informed consent and performed according to the Declaration of Helsinki. The women did not receive any significant financial benefit from their participation in the study and were asked to answer the questionnaires only once. The sample was found from three local medical clinics, from the departments of pediatrics and physiotherapy. The selection of our sample started in April 2024 and ended in July 2024, while the participants were asked to take part in the study anonymously. The electronic form of the questionnaires was distributed at the medical clinics that we collaborated with via Google Forms. The participants had open access to this form after being informed about the content of the questionnaires. The electronic form was completed anonymously by 71 women.

### 2.5. Statistical Analysis

Descriptive statistics were used to examine the demographic data of the sample. A normality test of the distribution of all variables was conducted using the Kolmogorov–Smirnov test. The Spearman’s rank correlation coefficient (Spearman r) was used to examine the correlations between the number of births and each factor of the ODQ and ICIQ- UI SF questionnaires, as well as between the total scores of the two questionnaires. The level of confidence was a = 0.05. Data analyses were performed using IBM SPSS Statistics for Windows Version 29.0.1.0 (ΙBM Corp., Armonk, NY, USA).

## 3. Results

The demographic data of the participants are summarized in Table 1. The number of children of the participating women ranged from 0 to 5. None of them had undergone surgery for UI and LBP symptoms, and only 2.8% of them were under medication for LBP. Before pregnancy, none of the women reported any symptoms of UI. Only one woman had menopause (1.4%).

According to the results, 22 women experienced UI during pregnancy (31%), and 20 women reported UI after childbirth (28.2%). In total, 69% of the participants reported that they “never have UI”, whereas 19.7% stated “usually once a week or less often” and 5.6% “several times per day”. Consequently, 4.2% (n = 3) of the participants described their condition as severe, 12.7% (n = 9) as moderate, and 83.1% (n = 59) as mild. Increased symptoms were observed in the first birth in women who underwent vaginal delivery, whereas in the second birth in women with caesarean section. In the third birth, there were increased symptoms of UI in women who had undergone cesarean section. Increased BMI (BMI 25–39.99 kg/m^2^) was associated with an increase in UI symptoms. The samples’ BMI categories were the following: underweight women 2.8% (n = 2), normal weight 56.3% (n = 40), overweight 35.2% (n = 25), and obesity with a rate of 5.7% (n = 4). Women who did not participate in any type of exercise experienced increased UI, with 66.7% (n = 2) of them appearing to have severe symptoms and 44.4% (n = 4) having moderate incontinence.

According to the ODQ score, 22.5% (n = 16) of the participants reported LBP before pregnancy, whereas 50.7% (n = 36) during pregnancy and 38% (n = 27) after childbirth. Chronic LBP appeared in 15.5% (n = 11). Women that underwent vaginal delivery in the first and second pregnancy have a higher score compared to women who underwent caesarean section. Women with one or two children also have high scores. When asked about weight lifting, 28.2% (n = 20) stated, “I can lift heavy objects, but it causes more pain”. Minimal LBP symptoms appeared in 93% (n = 66) of the women and moderate in 7% (n = 5). ODQ total score and BMI association revealed that higher score occurs in overweight women. Women under 30 years old in the first birth had lower scores, whereas the highest scores appeared in women 30–35 years old.

No correlation appeared between ICIQ-UI SF total score and number of births (r = 0.06, *p* = 0.61). The correlation between the total score of ODQ and the number of children was not statistically significant (r = −0.07, *p* = 0.53). No other significant correlations emerged between questionnaire factors and number of children. No correlation appeared between the total score of ODQ and BMI (r = −0.13, *p* = 0.26). Statistically significant correlations occurred between total scores of ICIQ-UI SF and ODQ (r = 0.33, *p* < 0.01) and ICIQ-UI SF total score and maternal age at first delivery (r = −0.26, *p* = 0.02) (Table 2).

## 4. Discussion

The aim of the present study was to examine the relationship between the number of children with the occurrence of UI and LBP. There was no association between LBP, UI, and the number of births according to the ODQ and ICIQ-UI SF. Similarly to Handa et al. [25], our study included women who had given birth for the first time in the last 5 years. Women who experienced UI during pregnancy and after delivery showed similar results, as it is reported that UI during pregnancy is a risk factor for its occurrence after delivery [34]. After childbirth, studies showed that vaginal delivery is associated with the occurrence of UI [2,12], while in the present study, vaginal delivery was associated with more symptoms of UI during the first delivery. Fewer symptoms of UI during the second birth occurred due to the lack of homogeneity between the samples’ numbers in the first and second birth. In the second birth, most women chose vaginal birth. No other study up until now has examined the relationship between the number of births and the occurrence of UI and LBP after pregnancy in a Greek population. In addition, in the present study, we recorded and examined the relationship between demographic variables, such as BMI, mothers’ age, height, and weight, with the total scores of the ODQ and ICIQ-UI SF questionnaires. No significant correlation emerged.

In particular, obesity and increased BMI (>25 kg/m^2^) are a considerable risk factor for UI [25,35]. In addition, Dai et al. [2] have identified as a significant risk factor for postpartum UI an increased mother’s BMI (≥24 kg/m^2^) before pregnancy. No statistically significant correlation of this variable was found in our study; however, the highest scores on the UI questionnaire were stated by the overweight women, while the lowest were stated by the underweight women. According to Ribeiro et al. [36], who stated that exercise is a way to prevent the occurrence of UI, increased symptoms of UI based on the total scores of ICIQ -UI SF were found in women who did not perform any type of exercise in the present study, but no correlation emerged. Performing pelvic floor exercises in early pregnancy and postpartum reduces the rate of UI in late pregnancy and postpartum [37].

According to the results of the present study, women who gave birth for the first time before 30 years old had the highest scores on the ICIQ UI-SF questionnaire, and women who gave birth at the age of 35 or older had the lowest scores. Contrary to our results, Dai et al. [2] and Ren et al. [3] reported that UI symptoms worsen depending on age during the first delivery, i.e., greater than or equal to 35 years old and 32 years old, respectively. The difference in the results of our study compared to the existing literature is due to the lack of homogeneity in the variable of age at first birth. The sample size in the second and third births was too small to consider important results, such as the increased symptoms of UI in women who had undergone cesarean section.

In the present study, no associations were found between ICIQ UI-SF and number of births. On the contrary, Wikander et al. [13] conclude that the number of births and mode of delivery affect the possibility of UI. The relationship between the number of births with urine incontinence after pregnancy has not been sufficiently studied in the Greek population.

No associations were found between ODQ factors and number of births. On the contrary, Heuch et al. [22] showed that having at least one delivery is associated with the risk of LBP, while there are no differences in symptoms between multiple deliveries. The largest percentage of women experienced LBP during pregnancy, while symptoms persisted in several women after delivery. The association of LBP after pregnancy with the number of births has not been sufficiently studied in the Greek population.

LBP has been examined mainly during and at the end of pregnancy, as it was associated with the weakness of the muscles in the area [16], while the present study focuses on postpartum symptoms. The relationship between LBP and BMI has not been adequately isolated and studied in women after pregnancy. However, it was reported that LBP after childbirth is not correlated with either BMI [38] or the number of children [8]. In our study, no correlation was observed between BMI and LBP. The group of women who gave birth for the first time before the age of 30 had the lowest scores on the questionnaire. On the contrary, Heuch et al. [22] reported that women under 20 years old at their first pregnancy are associated with a prevalence of chronic LBP.

Lack of physical activity is a predisposing factor for the occurrence of LBP in pregnant women [20]. A structured exercise program during pregnancy is effective in reducing the occurrence of pregnancy-related LBP and offers significant benefits to women’s quality of life [39]. Women who performed some type of exercise had fewer episodes of LBP compared to those who did not exercise regularly. Adding stabilization exercises to the daily routine of pregnant as well as postpartum women can prevent episodes of LBP in future pregnancies [40,41].

### Strengths, Limitations, and Future Recommendations

The major strength of the present study is that this is the first study that investigates a relationship between (i) the number of births and the occurrence of UI and LBP after pregnancy in a Greek population and (ii) demographical data with the occurrence of UI and LBP after pregnancy. Another strength is the utilization of previously tested and validated instruments that are used to assess UI and LBP. However, the study has some limitations. Firstly, there was no homogeneity in multiple births and the age at first birth. Secondly, the sample’s age, family status, and level of education differed. Thirdly, the entire sample was taken from a rural town, and it will be preferable in the future to use a larger and more homogenous sample.

Future research should be conducted to investigate the possible relationship between the number of children and UI symptoms in postpartum Greek women. It is important to carry out a more sophisticated statistical analysis, i.e., a regression analysis, for the examination between UI and LBP symptoms and the number of births in the Greek population. Future studies should examine the effect of the number of children on LBP symptoms, which has not been examined in the post-pregnancy Greek population, in order for the results to be more representative. In addition, more inclusion and exclusion criteria of the sample, such as prolapse, type of delivery, and family status, should be considered as they can affect the outcome of the symptoms. More information on the symptoms of UI and LBP is warranted for postpartum women. Future research should focus on how the different ages during their first birth affect UI symptoms. Further studies should be encouraged to examine, with larger samples, the relationship that appears between the total score of the incontinence questionnaire and the total score of the LBP questionnaire. More research should examine the symptoms of UI for each year up to 5 years postpartum and the mechanism that causes the symptoms of UI and LBP after childbirth in the Greek population.

## 5. Conclusions

Our results showed that there is no association between the number of children and the occurrence of UI symptoms five years after pregnancy. There was no correlation between the number of children and the symptoms of LBP after childbirth. Correlations appear between the total score of the incontinence questionnaire and that of the LBP, as well as between the total score of UI and age at first birth. Future research should be conducted with a larger and more homogeneous sample, to examine the effect between the number of children with the symptoms of UI and LBP postpartum in Greek women is important, as it contributes to reducing the postnatal effects in this population. Furthermore, it is important that similar studies should take place within an urban area. Further studies should investigate postpartum women who exercise in comparison to those who do not, tracking the improvement of UI and LBP symptoms.

## Figures and Tables

**Table 1:** Frequencies and percentages of the demographic data of the sample.

		Count	Table	Mean	Max	Min	Range	S.D.
**Age**		71	100.00%	34.99	45	24	21	4.31
**Weight**		71	100.00%	67.51	96	45	51	10.49
**Height**		71	100.00%	166.59	1.83	1.50	0.33	0.05
**B.M.I**		71	100.00%	24.26	35.15	17.68	17.47	3.66
**Exercise**	Yes	27	38.03%					
	No	44	61.97%					
**Education level**	Mandatory	2	2.81%					
	High school	13	18.31%					
	University	36	50.70%					
	Master Degree	20	28.16%					
**Family Status**	Married	70	98.60%					
	Divorced	1	1.40%					
**Professional Status**	Unemployed	9	12.68%					
	Employee	55	77.46%					
	Part-time job	7	9.86%					
**Age of first birth**	<30	29	40.84%					
	30 to <35	27	38.03%					
	>35 or =35	15	21.13%					
**First Birth**	Cesarean Section	36	50.70%					
	Vaginal Delivery	35	40.30%					
**Second Birth**	No birth	37	52.12%					
	Cesarean Section	14	19.72%					
	Vaginal Delivery	20	28.16%					
**Third Birth**	No birth	63	88.70%					
	Cesarean Section	5	7.00%					
	Vaginal Delivery	3	4.20%					

**Table 2:** Spearman’s r correlations between the scores of ICIQ and Oswestry Questionnaire and between age of first birth and ICIQ.

		Age at First Birth	Score ICIQ-UI SF	Score ODQ
**Age of first birth**	Correlation	1.00	−0.26 *	0.18
	Coefficient Sig (2-tailed)		0.02	0.13
	N	71	71	71
**Score ICIQ-SF UI**	Correlation	−0.26 **	1.00	0.33 **
	Coefficient Sig (2-tailed)	0.02		0.00
	N	71	71	71
**Score ODQ**	Correlation	0.18	0.33 **	1.00
	Coefficient Sig (2-tailed)	0.13	0.00	
	N	71	71	71

* Correlation is significant at the 0.05 level (two-tailed). ** Correlation is significant at the 0.01 level (two-tailed).

## Data Availability

The data presented in this study are available upon request from the corresponding author. The data are not publicly available due to internal regulations.
